# Supplementary material for: Super-Mitobarcoding in Plant Species Identification? It Can Work! The Case of Leafy Liverworts Belonging to the Genus Calypogeia
Source: Int J Mol Sci. 2022 Dec 8;23(24):15570. doi: 10.3390/ijms232415570 (PMC9779425; doi:10.3390/ijms232415570)
Supplement: Supplementary file 1 [file ijms-23-15570-s001.zip › ijms-2036772-supplementary/Supplementary_Material/Figure S1.pdf]

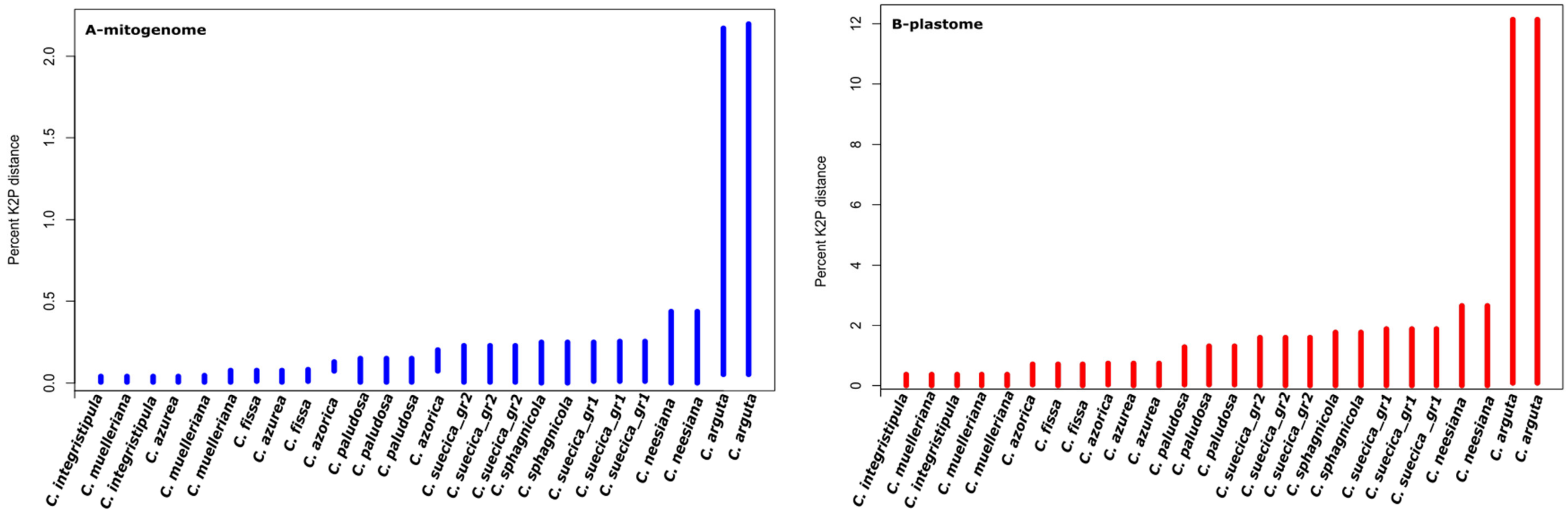

Figure S1. Line plot of the barcode gap for the *Calypogeia* species. (**A**) Data based on mitogenome. (**B**) Data based on the plastome. For each individual in the dataset, the lines represent the furthest intraspecific distance (bottom of line value), and the closest interspecific distance (top of line value).
