# Supplementary material for: Super-Mitobarcoding in Plant Species Identification? It Can Work! The Case of Leafy Liverworts Belonging to the Genus Calypogeia
Source: Int J Mol Sci. 2022 Dec 8;23(24):15570. doi: 10.3390/ijms232415570 (PMC9779425; doi:10.3390/ijms232415570)
Supplement: Supplementary file 1 [file ijms-23-15570-s001.zip › ijms-2036772-supplementary/Supplementary_Material/Table S2.pdf]

Table S2. Species used in this study, sampling data and sequencing results.

SPECIES	VOUCHER	geographic coordinates	sequencing results	MITOGENOME			PLASTOME		
				length [bp]	mean coverage	GenBank accession number	length [bp]	mean coverage	GenBank accession number
Calypogeia arguta	United Kingdom, coll. D.A.C.; DC1420	No data	Ślipiko et al. 2020 [52]	159,055	229.4	OM140682	Ślipiko et al. 2020 [52]		
	Spain, Galicia, Province Ourense, Serra Do Xurés National Park, coll. A.S.-V. and I.V.; S-V 31365	37.50°N, 25.48°W		159,061	181.2	MF401630			
Calypogeia integristipula	SE Poland, Bieszczady Mts, W slope of Mt Rozsypaniec Wołosacki, 1214 m, coll. K.B.; POZW 41928	49.06°N, 22.77°E		163,057	80.4	OM140683			
	S Poland, Tatra Mts, valley of Białka stream, 1100 m a.s.l., coll. K.B., A.B.; POZW 40856	49.24°N, 20.10°E		163,057	143.7	MF401629			
Calypogeia fissa	W Poland, Lubuskie Province, Biecz forestry, coll. S.R., K.B.; POZW 42306	51.48°N, 15.10°E		162,174	169.1	OM117689			
	NW Poland, Pomorskie Province, Lake Małe Sitno near Czarna Dąbrówka, coll. K.B., A.B. POZW 42345	54.16°N, 17.31°E		162,175	199.1	MF401632			
Calypogeia suecica gr.1	SE Poland, Bieszczady Mts, Wetlina, valley of Średni Lutowy stream, on soil, 640 m a.s.l., coll. K.B.; POZW41937	49.12°N, 22.47°E		160,451	89.0	OM140680	Ślipiko et al. 2020 [52]		

SPECIES	VOUCHER	geographic coordinates	sequencing results	MITOGENOME			PLASTOME		
				length [bp]	mean coverage	GenBank accession number	length [bp]	mean coverage	GenBank accession number
	SE Poland, Bieszczady Mts, Wetlina, valley of Górna Solinka stream,, 680 m a.s.l., coll. K.B. POZW41930	49.14N, 22.48E	**19,325,216 paired 150bp reads**	160,433	44.9	OP526745	**119,977**	120.1	OP526752
	S Poland, Tatry Mts, NE slope of Skupinów Upłaz Mt, 1200 m a.s.l., coll. K.B., A.B. POZW43102	49.26N, 19.99E	**19,359,240 paired 150bp reads**	160,440	118.3	OP526748	**119,960**	304.5	OP526753
Calypogeia suecica gr.2	SE Poland, Beskid Sądecki Mts, Potok Czarny stream, 717 m, coll. K.B. POZW 42366	49.26°N, 20.28°E	Ślipiko et al. 2020 [52]	161,960	101.7	NC_035979	Ślipiko et al. 2020 [52]		
	S Poland, Pieniny Mts, valley of Limbargowy stream, 760 m a.s.l., coll. K.B. POZW43100	49.42N, 20.37E	**18,033,798 paired 150bp reads**	161,975	65.4	OP526747	**120,116**	199.8	OP526750
	SE Poland, Bieszczady Mts, Wetlina, valley of Górna Solinka stream, 772 m a.s.l., coll. K.B. POZW41936	49.14N, 22.48E	**18,487,662 paired 150bp reads**	161,975	78.4	OP526746	**120,117**	233.1	OP526751
Calypogeia neesiana	S Poland, Tatra Mts, N slope of Mt Ornak, 1680 m, coll. K.B., A.B. POZW 41731	49.13°N, 19.50°E	Ślipiko et al. 2020 [52]	163,324	92.3	OM140681	Ślipiko et al. 2020 [52]		
	SE Poland, Bieszczady Mts, W slope of Mt Tarmica, 1280 m, coll. K.B.; POZW 41952	49.05°N, 22.44°E		163,324	41.2	OP554114			
Calypogeia azurea	S Poland, Tatra Mts, NE slope of Skupinów Upłaz	49.27°N, 19.99°E		162,031	192.4	OM140676			

SPECIES	VOUCHER	geographic coordinates	sequencing results	MITOGENOME			PLASTOME		
				length [bp]	mean coverage	GenBank accession number	length [bp]	mean coverage	GenBank accession number
	Mt, 1200 m, coll. K.B., A.B.; POZW 41372		Ślipiko et al. 2020 [52]				Ślipiko et al. 2020 [52]		
	S Poland, Tatra Mts, Rów Zakopiański at N base of Tatra Mts, 971 m, coll. A.B., K.B.; POZW 41388	49.31°N, 20.05°E		162,033	105.7	OM140675			
Calypogeia sphagnicola	NW Poland, Pomorskie Province, Lake Wałachy near Kościerzyna, coll. K.B., A.B.; POZW 42243	54.00°N, 17.57°E		160,890	36.5	OM140679			
	S Poland, Row Zakopiański at N base of Tatra Mts, 971 m, coll. K.B., A.B.; POZW 41695	49.31°N, 20.05°E		160,890	50.1	OP526749			
Calypogeia paludosa	S Poland, Tatra Mts, lake Toporowy Staw Wyżni, coll. K.B., A.B. POZW 41142	49.16°N, 20.01°E		162,096	177.1	OM140674			
	S Poland, Tatra Mts, E slope of Mt. Żołta Turnia, 1,687 m, coll. K.B., A.B. POZW 41178	49.14°N, 20.00°E		162,096	77.8	OM140673			
	S Poland, Tatra Mts, Pańszczyca Valley, peat bog Wielka Pańszczycka Młaka, 1,274 m a.s.l. K.B., A.B.; POZW 41173	No data		162,126	65.6	OM140671			
Calypogeia muelleriana	W Poland, Lubuskie Province, Biecz forestry, coll. S.R., K.B. POZW 42318	51.48°N, 15.10°E		162,842	10,078.6	OM140672			

<table>
<tr><th rowspan="2">SPECIES</th><th rowspan="2">VOUCHER</th><th rowspan="2">geographic coordinates</th><th rowspan="2">sequencing results</th><th colspan="3">MITOGENOME</th><th colspan="3">PLASTOME</th></tr>
<tr><th>length [bp]</th><th>mean coverage</th><th>GenBank accession number</th><th>length [bp]</th><th>mean coverage</th><th>GenBank accession number</th></tr>
<tr><td rowspan="2"></td><td>NW Poland, Pomorskie Province, Lake Orle near Miastko, coll. K.B., A.B. POZW 41346</td><td>54.01°N, 17.04°E</td><td rowspan="4">Ślipiko et al. 2020 [52]</td><td>162,913</td><td>48.8</td><td>OM140669</td><td colspan="3" rowspan="4">Ślipiko et al. 2020 [52]</td></tr>
<tr><td>NW Poland, Pomorskie Province, Lake Lubygość near Kartuzy, coll. K.B., A.B. POZW 42220</td><td>54.24°N, 17.59°E</td><td>162,909</td><td>100.8</td><td>OM140670</td></tr>
<tr><td rowspan="2">Calypogeia azorica</td><td>Portugal, Azores, Sao Migiel Island, coll. A.S.-V. and I.V. S-V 29154</td><td>37.50°N, 25.48°W</td><td>161,851</td><td>176.2</td><td>OM140677</td></tr>
<tr><td>Portugal, Azores, Sao Migiel Island, coll. A.S.-V. and I.V. S-V 29425</td><td>37.44°N, 25.18°W</td><td>161,848</td><td>69.9</td><td>OM140678</td></tr>
</table>

Collectors: A.B. – Alina Bączkiewicz, A.S. – A. Sloga, A.S.-V. – Alfons Schäfer-Verwimp, I.V. – I. Verwimp, K.B. – Katarzyna Buczkowska, D.A.C. – Des A. Callaghan, D.Q. – D. Quandt, S.R. – Stanisław Rosadziński; **Herbaria:** DC – Herb. D. A. Callaghan, POZW – Herbarium of Adam Mickiewicz University, S -V – Herb. Schäfer-Verwimp
